# Experimental Evaluation of Aerosol Production after Dental Ultrasonic Instrumentation: An Analysis on Fine Particulate Matter Perturbation

**DOI:** 10.3390/ijerph18073357

**Published:** 2021-03-24

**Authors:** Filippo Graziani, Rossana Izzetti, Lisa Lardani, Michele Totaro, Angelo Baggiani

**Affiliations:** 1 Department of Surgical, Medical and Molecular Pathology and Critical Care Medicine, University of Pisa, 56126 Pisa, Italy; rossana.izzetti@med.unipi.it (R.I.); lisa.lardani@gmail.com (L.L.); 2 Sub-Unit of Periodontology, Halitosis and Periodontal Medicine, University Hospital of Pisa, 56126 Pisa, Italy; 3 Hygiene and Epidemiology Unit, Department of Translational Research and the New Technologies in Medicine and Surgery, University of Pisa, 56126 Pisa, Italy; michele.totaro.unipi@hotmail.com (M.T.); angelo.baggiani@med.unipi.it (A.B.)

**Keywords:** aerosols, particulate matter, dental scaling, occupational exposure, air pollution, indoor, air quality

## Abstract

Aerosol production represents a major concern during the majority of dental procedures. The aim of the present study is to investigate the dynamics of aerosol particles after 15 min of continuous supragingival ultrasonic instrumentation with no attempt of containment through particle count analysis. Eight volunteers were treated with supragingival ultrasonic instrumentation of the anterior buccal region. A gravimetric impactor was positioned 1 m away and at the same height of the head of the patient. Particles of different sizes (0.3–10 µm) were measured at the beginning of instrumentation, at the end of instrumentation (EI), and then every 15 min up to 105 min. The 0.3-µm particles showed non-significant increases at 15/30 min. The 0.5–1-µm particles increased at EI (*p* < 0.05), and 0.5 µm remained high for another 15 min. Overall, all submicron aerosol particles showed a slow decrease to normal values. Particles measuring 3–5 µm showed non-significant increases at EI. Particles measuring 10 µm did not show any increases but a continuous reduction (*p* < 0.001 versus 0.3 µm, *p* < 0.01 versus 0.5 µm, and *p* < 0.05 versus 1–3 µm). Aerosol particles behaved differently according to their dimensions. Submicron aerosols peaked after instrumentation and slowly decreased after the end of instrumentation, whilst larger particles did not show any significant increases. This experimental study produces a benchmark for the measurement of aerosol particles during dental procedures and raises some relevant concerns about indoor air quality after instrumentation.

## 1. Introduction

The risk assessment of pathogenic transmission through dental aerosols has been repeatedly discussed over the years due to the relevant aerosol production during the majority of dental procedures [1,2]. The current diffusion of the SARS-CoV-2 pandemic has highlighted the need for a better understanding of the dynamics and potential infectivity of dental aerosols [3].

Airborne infection transmission occurs through the persistence of a suspension of fine liquid droplets or solid particles in air or in another gas. Infected aerosols (bioaerosols) are characterized by the presence of either dead or live microorganisms, which are responsible for the development of various adverse health outcomes. Exposure to bioaerosols can cause infections, as both bacteria, including *Mycobacterium* spp., *Pseudomonas* spp., *Legionella* spp., *Staphylococcus aureus*, and *Streptococcus* spp., and viruses, such as rhinovirus, HIV, HBV, HCV, and herpes viruses, may be carried through airborne particles [4,5]. Moreover, the development of allergies, immune reactions, non-allergic inflammations, and toxic effects may also be related to aerosol exposure [6]. The concerns regarding bioaerosols were previously raised with the advent of severe acute respiratory syndrome (SARS), Middle Eastern respiratory syndrome (MERS), and Ebola [7]. All of the aforementioned conditions are characterized by a predominantly airborne diffusion, which exposes both the general population and health care workers to a high risk of contagion.

Aerosols can be classified according to the dimensions of the particles involved [8,9]. This has an impact on the potential access to the lower respiratory tract. In particular, particles >10 µm are blocked in the nasal region, while 5–10 µm particles can reach and deposit in the upper respiratory system [8,9]. If the aerodynamic diameter of the particle is smaller than 5 µm, aerosol particles can reach the pulmonary alveoli and cause lower respiratory tract infection [9,10]. Transmission of airborne particles can be thus classified as (1) droplet transmission, occurring via direct contact of droplets with oral, nasal, and eye mucosa or through direct inhalation, and (2) aerosol transmission, where airborne particles <5 µm remain suspended in the air and reach the lungs through inhalation.

As recently claimed [11], dental professionals appear to be among the most exposed health care workers to aerosols due to the high number of aerosol-generating procedures performed. In the dental setting, aerosol levels show an exponential increase when using ultrasonic scalers, high-velocity rotating handpieces, and three-in-one water syringes [12,13,14,15]. Moreover, the high microbial load of dental aerosols derived from saliva, blood, nasopharyngeal secretions, plaque, calculus, and dental materials contributes to the risk of infection in dental health care workers [16].

Although current literature reports that dental procedures generate a large amount of aerosols, the heterogeneity of the methodologies applied in terms of sampling, study setting, and the particular attention towards the microbiological impact of aerosols hinders the possibility to compare previous estimations of bioaerosol profile [17]. Moreover, there is a lack of systematic investigation regarding the aerodynamic diffusion of aerosols and their persistence in suspension in the dental setting. It is therefore of utmost importance to understand the dynamics of aerosols generated during dental procedures, in particular the periodontal ones, which appear to be associated with a significant aerosol production. 

The aim of this study is therefore to investigate the pattern of production, diffusion, and persistence of aerosols as measured through the analysis of particle concentration produced during ultrasonic instrumentation.

## 2. Materials and Methods

### 2.1. Experimental Design and Study Setting 

This was a single-center prospective evaluation of aerosol production on healthy volunteers with no measures of aerosol containment taken. The study was approved by the Committee on Bioethics of the University of Pisa (Review No. 22/2020) and was conducted according to the principles outlined in the Declaration of Helsinki on experimentation involving human subjects. Prior to the study beginning, study participants were given detailed information on the investigation being performed and signed an informed written consent form in the case of acceptance to participate in the study.

Inclusion criteria were (1) adults ≥18 years of age, (2) apparent good health status, and (3) acceptance to be included in the study. Exclusion criteria were (1) pregnancy or breastfeeding, (2) any acute or chronic condition that would limit the ability of the patient to participate in the study, and (3) refusal to give informed consent. 

The study was performed from September to October 2020 in two dental operating rooms (A and B) of similar dimensions (40 square meters) and comparable in terms of ventilation (absent), temperature (21 °C), and relative humidity (85%) as assessed by means of data loggers. The participants were randomly assigned to one of the two operating rooms using a computer-generated table created by an experimenter not directly involved in the study. During all experiments, the room temperature was constant. Air conditioning and ventilation systems were switched off during the experimental analysis, and windows were kept closed in order to avoid potential interference with the experimental environment. 

### 2.2. Study Testing

The study participants received supragingival ultrasonic scaling (EMS, Nyon, Switzerland) on the buccal surfaces of the anterior area on both the mandible and maxilla (canine to canine). The dental chair was reclined (patient’s head below the operator’s elbow), and the operator was sitting at the right side of the patient (9 o’ clock position). A single aspirator was used and placed in the inferior retro-molar trigone, but no additional measures to prevent aerosol production were adopted. Instrumentation lasted 15 min exactly in all tests. No subjects were allowed in the rooms other than the operator and the volunteer during testing. During instrumentation, the dental chair was reclined completely. 

### 2.3. Particle Count

A gravimetric impactor (Hach Met One 3313 Particle Counter, Ashtead Technology, Sandy, UK) was employed for the evaluation of the particle number concentration (P_num_), i.e., the number of particles within a given volume (particles/cm^3^), the sampling of aerosol particles in the range between 0.3 and 10.0 µm (standard size channels of 0.3, 0.5, 1.0, 3.0, 5.0, and 10.0 µm). The gravimetric impactor had a fixed flow rate of 28.3 LPM (1.0 CFM) ±5% (default factory setting). Counting efficiency data are 50 ± 20% for 0.3 and 0.5 µm, (100 ± 10% at 1.5 times the minimum sensitivity), fully complying with ISO 21501-4. Before the tests were performed, the particles were not dried in order to simulate the real environmental conditions.

The impactor was placed at the same height of the volunteer’s head and positioned 1 m away, opposite to the operator. Thus, sampling particulate matters of 0.3, 0.5, 1, 3, 5, and 10 µm were assessed. All of the measurements were performed in triplicate on three separate occasions.

P_num_ assessment was performed every 15 min. An initial registration was performed as a baseline assessment; then, 15 min afterwards, instrumentation testing began (beginning of instrumentation - BI). At the end of instrumentation (EI), measurements were taken again. At EI, all of the researchers left the room and periodic registrations were made every 15 min up to 120 min after BI (105 min after EI).

### 2.4. Sample Size Estimation

The aim was to evaluate the variation of the aerosol particle concentration over time. To achieve this result, sample size calculation indicated that a minimum of 6 registrations for each particle group (0.3, 0.5, 1, 3, 5, and 10 µm) at 10 time points were needed to determine a difference between groups (*p* < 0.05, α = 0.90). 

### 2.5. Data Analysis

All data are presented as mean and standard deviation unless otherwise specified. Data were tested for normality, and logarithmic or square root transformations in P_num_ values and were produced as needed before being applied to the appropriate testing.

Changes in all P_num_ were analyzed using ANOVA for repeated measures between groups at different time points. Only significant differences versus baseline, BI, EI, and 15 min are reported for practical reasons. For P_num_ with a statistically significant difference versus baseline, a percentual relative increase at the end of the treatment and 15 min and a relative decrease at 60 min were calculated as follows: P_num_ (end of instrumentation, 30 min, or 60 min) minus P_num_ (beginning of instrumentation) divided by P_num_ (beginning of instrumentation) and multiplied by 100. Comparison of the relative increase/decrease groups was performed by t-test or an equivalent non-parametric method. 

Confidence interval was set at 95% (*p* < 0.05). All analyses were performed with SPSS version 23 (SPSS Inc. Chicago, IL, USA).

## 3. Results

**Figure 1:**
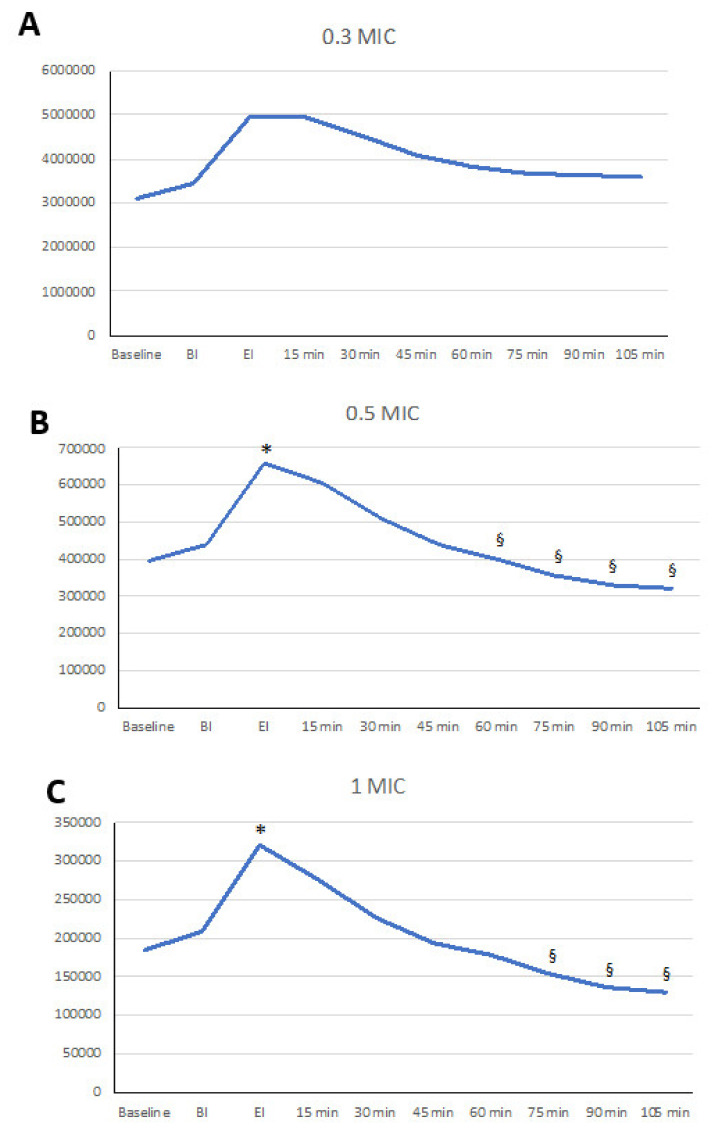

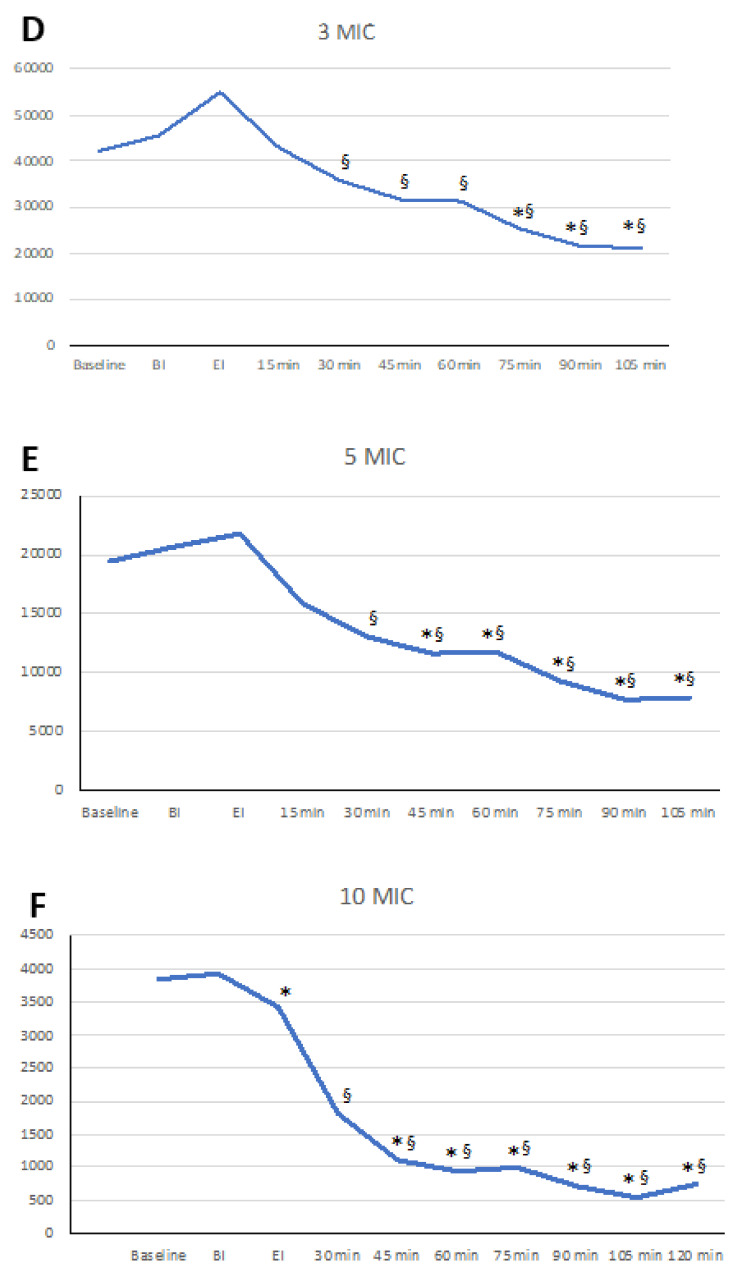
Linear graphs representing the dynamics of the aerosols after ultrasonic scaling. On the vertical axis, particle number is represented. Significant differences versus beginning of instrumentation - BI (*) and end of instrumentation - EI (§) are marked. (**A**) P_num_ of 0.3 µm; (**B**) P_num_ of 0.5 µm; (**C**) P_num_ of 1.0 µm; (**D**) P_num_ of 3.0 µm; (**E**) P_num_ of 5.0 µm; (**F**) P_num_ of 10.0 µm.

**Figure 2:**
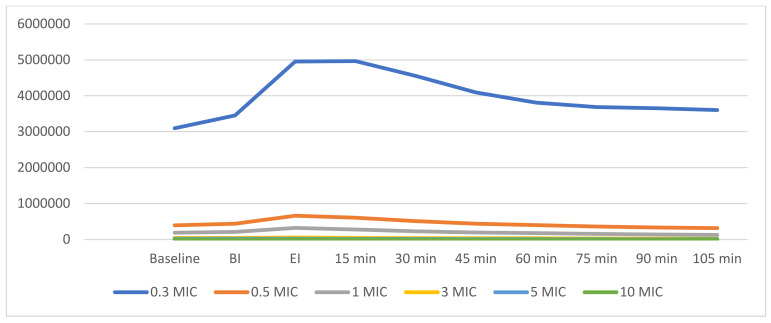
Linear graphs representing the dynamics of aerosols after ultrasonic scaling. Summary of aerosol dynamics of differently sized particles. MIC = abbreviation for µm

In total, eight patients were recruited (4 M, 4 F, mean age 33.75 ± 6.85). All of the study participants completed the study. During the experimentation, no adverse events or compliance issues were recorded. Baseline values identified values within the range expected in terms of air cleanliness by particle concentration (ISO 14644-1:2015) in both rooms. In Figure 1 and Figure 2, the variations of particle concentrations are shown.

### 3.1. Submicron and Micro Aerosol

Changes in P_num_ of 0.3 µm through time did not show significant changes, although a trend was identified with 0.3 µm particles increasing at EI and 15 min afterwards. After 30 min, a mild reduction was observed (Table 1, Figure 1A).

Particles of 0.5 µm showed a significant increase at EI and 15 min afterwards compared to baseline and at the beginning of instrumentation (*p* < 0.05), showing an increase of 81% (st. dev 88%) in baseline values at EI and 58% (st. dev 67%) at 15 min. At 60 min and onwards, significant differences among EI and the 15 min assessments were observed (Figure 1B, Table 1). 

Particles of 1 µm showed a significant increase at the end of instrumentation and 15 min afterwards compared to baseline and the beginning of instrumentation (*p* < 0.05). of 95% (st. dev 98%). This peak decreased significantly at 60 min and 75 min (*p* < 0.05) and even more so at 90 min (*p* < 0.01) and 120 min (*p* < 0.001) (Figure 1C, Table 1). 

No statistically significant differences were noted among these particles in terms of percentage increase at EI and 15 min nor in terms of the decrease at 60 min. 

### 3.2. Aerosol Particles >3 µm 

Particles of 3 and 5 µm did not show any significant variations after instrumentation (Figure 1D,E, Table 2). Overall, the larger the particle was, the lower the EI increase, as 64% (st. dev 79%) and 41% (st. dev 65%) for 3 and 5 µm, respectively, were observed. Values at EI were significantly different from values observed at 45 min up to 120 min (*p* < 0.0001 for all). 

Particles of 10 µm did not show any increase, but a continuous decrease that appeared to be more intense than that observed in all of the other particles as measured with a percent decrease of −66% (st. dev 26%) at 60 min (*p* < 0.001 versus 0.3 µm, *p* < 0.01 versus 0.5 µm, and *p* <0.05 versus 1 and 3 µm) (Figure 1F, Table 2).

## 4. Discussion

In this study, aerosol production was measured through particulate matter analysis. Ultrasonic instrumentation determined some relevant perturbation of the indoor air quality that is of interest. In particular, smaller particles tended to show peaks after instrumentation and then a steady, yet slow, decrease. On the other hand, larger particles did not show an increase or showed a notable decrease after instrumentation.

The exposure to contaminated aerosols represents the second most frequent cause of infection among hospital care workers [18]. The total bacterial bioaerosol concentrations are reported to reach extremely high levels (up to 77 cfu/m^3^) in hospital inpatient facilities, while in the dental setting, more than 38 different bacterial and fungal species have been identified in bioaerosols [17]. Some previous studies reported different methods for the evaluation of aerosols generated by dental procedures [12,19,20,21]. The evaluation of bacterial colony-forming units on agar plates was the most frequently adopted technique for the estimation of bioaerosol production, although other techniques, such as dark-field microscopy, Gram stain microscopy, and chemical identification, have also been employed. Bennett et al. [19] reported that higher levels of oral microorganisms were generated during scaling, but in a period of 10–30 min, aerosol peaks returned to baseline values. 

Our data indicate that fine aerosols, measured through P_num_ of particles in the range 3–10 μm, did not have significant variations after instrumentation. Conversely, a steady decrease was observed from 45 to 120 min, as it is assumed that larger particles are heavier and therefore would have a higher precipitation than smaller particles [9]. This result is consistent with the observation that particles in the 10–100 µm size range have inertial velocities comparable to gravitational settling, and that the suspension velocities are quite similar to the deposition velocities for particles in the 10–50 µm size range, thus showing ballistic behavior [22]. Particle numbers decreased below the baseline after 105 min. This result could be explained as a return to the initial conditions of the room prior to the entrance of the experimenters for the preparation of the experimental setting, which could have caused slight initial perturbation.

At baseline, the room was populated for different activities. This allowed the particles to move from the surfaces to the air.

On the other hand, in our study, submicron and micro-particulate matter significantly increased after 15 min of continuous ultrasonic instrumentation. The extent of the increase was approximately double the baseline values, indicating an important perturbation within one meter of distance to the patient. According to the data, ultrasonic instrumentation can accurately detect some aerosols, especially those of smaller particles. On the other hand, particles tend to remain airborne for variable periods depending on their diameter. Smaller particles are characterized by their longer persistence in the air compared to larger droplets (Ø >5 µm), which present a relatively fast precipitation [17,18,23]. Indeed, Dutil et al. [24] investigated the culturable airborne bacterial concentration after 30 min of dental cleaning with an ultrasonic scaler, indicating that bioaerosol concentration increased during treatment with a median of 2800 CFU/m^3^, and that it was predominantly composed of small particles.

The behavior of aerosol particles may be described as a sequence of phases including the deagglomeration and the settling phase [25,26]. In a closed and unventilated room, particles of the same size will settle at the same speed in still or stagnant air. Considering that the largest particles decay by settling, the absence of air flow does not exclude the occurrence of particle deagglomeration [25,26]. This phenomenon causes the increase in smaller particles after several minutes in the presence of air stagnation. After a few hours of this condition, the impact of smaller particles on room surfaces can also no longer be detected by the gravimetric impactor [25,26].

Interestingly, P_num_ values return to the beginning of instrumentation levels 30–45 min after instrumentation. This indicates that a “nebula” of submicron particles remains floating in the proximity of the chair, as has already been noted [16,19]. This is of utmost importance, as the presence of SARS-CoV-2 is also reported in the particles ranging between 0.25 and 1.0 μm [27]. Thus, theoretically, a bioaerosol carrying viruses might remain within the proximity of the dental chair even after the patient leaves. 

The potential biological hazard of this “nebula” of submicron aerosols has been supposed but not demonstrated. This should represent the basis of the rationale of the supposedly higher risk category in which oral health care providers would fall. Nevertheless, recent reports from the Lombardy region, i.e., the Italian area with the highest level of COVID-19 outbreak, do not suggest a higher incidence than that of the general population, as the 0.86% were eventually proven to be positive for SARS-CoV-2 [28]. This would then suggest that dentists and their staff are more protected than general doctors, indirectly suggesting that the average protection measures might provide a sufficient level of protection. Moreover, it might also be speculated that viral concentration in ultra-fine aerosols may be extremely variable in dental practices. 

It is also important to raise awareness on the issue that, irrespective of the theoretical risk of SARS-CoV-2 transmission, indoor air quality is of utmost importance for health care workers [29,30,31]. Indoor air quality has a significant impact on health and quality of life in general [32]. Indoor environments are a mélange of outdoor and indoor contaminants. Outdoor pollutants enter through infiltrations and natural/mechanical ventilation systems. Indoor contaminants are the result of emissions from combustion, materials and furnishings, heating/cooling/humidification systems, electronic equipment, cleaning products, and the occupants’ behavior [32]. Indoor air quality is affected by gases; volatile organic compounds; particulate matter; organic and inorganic contaminants; and biologicals, such as bacteria and pollen [33]. Indoor air pollution has been associated with asthma or allergy attacks, headaches, nausea, and irritation of the eyes, nose, and throat [33].

To the best of our knowledge, this is the first study to analyze the dynamics of production, diffusion, and precipitation of airborne particles in the dental setting. In this proof-of-concept study, the characteristics of the aerosols produced through ultrasonic scaling were investigated and highlight the need for further evaluation regarding other dental procedures at risk of producing a high number of aerosolized particles, possibly using larger study samples. However, only a physical analysis of the aerosols was provided, and the microbiological aspect of the bioaerosols was not evaluated at this stage of the research. Moreover, due to a technical limitation of the gravimetric impactor employed, it was not possible to sample particles smaller than 0.3 µm. The creation of smaller particles due to impaction of larger particles is a real phenomenon, and it could explain why the particle number showed a higher increase for the smaller particles as opposed to the larger particles at EI. We can thus hypothesize that the presence of small particles below the sampling capability of the instrument may have contributed to this result. Finally, an integration of physical and microbiological data may provide additional information on the actual risk of infection related to bioaerosol production.

The long viability of SARS-CoV-2 in air for up to 3 h [34] highlights the need for a deeper understanding of the dynamics of the aerosols produced during dental procedures. Moreover, modeling the dynamics of the aerosols generated during dental procedures may expand our knowledge regarding not only the timely issue of COVID-19 but also other airborne transmitted infectious diseases, and it may provide some important insights into the evaluation of indoor air quality. These data should provide a benchmark to evaluate other types of instrumentation and, most importantly, actions to be taken to prevent aerosol formation. 

## 5. Conclusions

This is the first study reporting on the dynamics of aerosol production following ultrasonic scaling. The 0.3-µm particles did not show significant changes, although increasing at EI and at 15 min. The 0.5-µm particles increased by 81% at EI and 15 min compared to baseline values and by 58% at 15 min, while 1-µm particles showed a peak at 15 min and a decrease that was observed up to 120 min afterwards. Larger particles (>1 µm) did not vary significantly during the observation time. The behavior of 0.5–1-µm particles appears of interest in the assessment of indoor air quality.

## Figures and Tables

**Table 1:** Means and standard deviations of particles of submicron and micro aerosols throughout the study. Table legend. * *p* < 0.05 vs. baseline; § *p* < 0.05 vs. BI; # *p* < 0.05 vs. the end of instrumentation (EI) min; ## *p* < 0.01 vs. EI min; ° *p* < 0.05 vs. 15 min; °° *p* < 0.01 vs. 15 min.

	0.3 μm		0.5 μm		1 μm	
	Mean	Std. Deviation	Mean	Std. Deviation	Mean	Std. Deviation
BASELINE	3,099,087	1,874,625	395,197	198,491	185,647	98,743
BI	3,453,958	2,133,727	439,378	217,760	209,509 *	104,464
EI	4,952,485	3,012,991	660,629 *§	360,456	321,844 *§	189,350
15 min	4,968,704	2,720,239	607,357 *	280,691	276,460	131,833
30 min	4,551,001	2,439,808	508,893	217,209	226,759	102,712
45 min	4,092,890	2,018,861	439,127	151,974	194,071	68,837
60 min	3,807,691	1,799,915	400,761 #	113,393	178,676 #	58,406
75 min	3,683,709	1,792,499	357,687 °#	94,795	153,199 ##°	44,556
90 min	3,649,827	1,703,106	330,118 ##°°	86,997	135,673 ##°	38,993
105 min	3,604,367	1,587,125	320,151 ##°°	74,592	129,292 ##°°	33,588

**Table 2:** Means and standard deviations of particles of extremely and ultra-fine aerosols throughout the study. Table legend. * *p* < 0.05 vs. baseline; ** *p* < 0.01 vs. baseline; *** *p* < 0.001 vs. baseline; § *p* < 0.05 vs. BI; §§ *p* < 0.01 vs. BI; §§§ *p* < 0.001 vs. BI; # *p* < 0.05 vs. EI min; ## *p* < 0.01 vs. EI min; ### *p* < 0.01 vs. EI min; ° *p* < 0.05 vs. 15 min; °° *p* < 0.01 vs. 15 min; °°° *p* < 0.001 vs. 15 min.

	3 μm		5 μm		10 μm	
	Mean	Std. Deviation	Mean	Std. Deviation	Mean	Std. Deviation
BASELINE	42,153	24,121	19,433	10,202	3839	1939
BI	45,760	24,418	20,775	11,565	3928	2651
EI	54,873	16,593	21,806	6069	3423 *	1586
15 min	43,024	12,333	15,935	4790	1821 ***§#	979
30 min	35,822 #	12,094	13,039 #	4136	1110 ***§§§###	408
45 min	31,508 ##	10,067	11,593 §#	4131	930 ***§§§###°°	345
60 min	31,238 ##	15,181	11,732 *§§##	6448	1002 ***§§§###°°	580
75 min	25,409 §##°	10,438	9341 **§§§###°°	4365	717 ***§§§###°°°	423
90 min	21,408 §§**###°°	7708	7698 ***§§§###°°	3068	546 ***§§§###°°	272
105 min	21,011 §§**###°°	7359	7846 ***§§§###°°°	3321	726 ***§§§###°°°	450

## Data Availability

Data are contained within the article.
